# Involvement of the reward network is associated with apathy in cerebral small vessel disease

**DOI:** 10.1016/j.jad.2018.02.006

**Published:** 2018-05

**Authors:** Danuta M. Lisiecka-Ford, Daniel J. Tozer, Robin G. Morris, Andrew J. Lawrence, Thomas R. Barrick, Hugh S. Markus

**Affiliations:** a Stroke Research Group, University of Cambridge, Department of Clinical Neurosciences, Cambridge, UK; b King's College London, Institute of Psychiatry, Psychology and Neuroscience, London, UK; c St. Georges, University of London, Neurosciences Research Centre, London, UK

**Keywords:** SVD, small vessel disease, MRI, magnetic resonance imaging, WMH, white matter hyperintensities, DTI, diffusion tensor imaging, VTA, ventral tagmental area, SCANS, St George's Cognition and Neuroimaging in Stroke, GDS, Geriatric Depression Scale, SPGR, spoiled gradient recalled echo, FSL, FMRIB Software Library, AAL, Automated Anatomical Labelling, Apathy, Reward, Neural network, Graph theory, Small vessel disease, Lacunar stroke

## Abstract

**Introduction:**

Apathy is a common yet under-recognised feature of cerebral small vessel disease (SVD), but its underlying neurobiological basis is not yet understood. We hypothesized that damage to the reward network is associated with an increase of apathy in patients with SVD.

**Methods:**

In 114 participants with symptomatic SVD, defined as a magnetic resonance imaging confirmed lacunar stroke and confluent white matter hyperintensities, we used diffusion tensor imaging tractography to derive structural brain networks and graph theory to determine network efficiency. We determined which parts of the network correlated with apathy symptoms. We tested whether apathy was selectively associated with involvement of the reward network, compared with two “control networks” (visual and motor).

**Results:**

Apathy symptoms negatively correlated with connectivity in network clusters encompassing numerous areas of the brain. Network efficiencies within the reward network correlated negatively with apathy scores; (r = − 0.344, p < 0.001), and remained significantly correlated after co-varying for the two control networks. Of the three networks tested, only variability in the reward network independently explained variance in apathetic symptoms, whereas this was not observed for the motor or visual networks.

**Limitations:**

The analysis refers only to cerebrum and not cerebellum. The apathy measure is derivative of depression measure.

**Discussion:**

Our results suggest that reduced neural efficiency, particularly in the reward network, is associated with increased apathy in patients with SVD. Treatments which improve connectivity in this network may improve apathy in SVD, which in turn may improve psychiatric outcome after stroke.

## Highlights

• Apathy is an important affective consequence of cerebral small vessel disease.
• Our study investigates neuromechanism of apathy in small vessel disease.
• We show that apathy is associated with structural disconnectivity in the reward network.

## 1 Introduction

Cerebral small vessel disease (SVD) affects the small arteries of the brain supplying the white matter and deep grey matter nuclei of the brain. It results in pathological changes in the brain tissue and characteristic radiological features best detected using magnetic resonance imaging (MRI), including lacunar infarcts, T2-white matter hyperintensities (WMH), cerebral microbleeds, and more diffuse white matter damage measured using diffusion tensor imaging (DTI). SVD results in a characteristic cognitive profile with early stage specific impairment in executive function and information processing speed (Lawrence et al., 2013). The psychological changes in SVD, it has been suggested, result from a disconnection syndrome with white matter tracts underlying complex subcortical-cortical circuits disrupted by the different neuropathologies. This is supported by recent findings from MRI structural network analysis (Lawrence et al., 2014).

In addition to cognitive impairment, apathy is a common affective feature of SVD (Brodaty et al., 2005). Apathy comprises lack of motivation, behavioural and cognitive retardation and reduced emotional reactivity and it results in diminished goal-oriented behaviour (Marin, 1996). It is a prominent affective symptom of many neurological diseases. The neurobiological mechanisms underlying apathy in SVD are not yet fully understood. Some studies report the volume of WMH as a predictor of apathy in SVD, (Grool et al., 2012, Gupta et al., 2014, Nebes et al., 2001, Yao et al., 2015, Yao et al., 2009) but this has not been confirmed in further studies (Cosin et al., 2015, Grool et al., 2013). In a recent study apathy in SVD was correlated with decreased DTI fractional anisotropy in the cingulum, fornix and uncinate fasciculus, suggesting that white matter damage in these brain regions is more specifically causal (Hollocks et al., 2015). A recent review has identified more broadly diminished connectivity in frontal, subcortical and parietal areas as associated with apathy across many neurological and psychiatric conditions (Moretti and Signori, 2016). In SVD it has been hypothesized that reduction in white matter integrity affects the efficiency of reward networks which in turn results in apathy (Quattrocchi and Bestmann, 2014).

The reward network may play an important role in occurrence of apathy due to its role in the motivational process (Murayama et al., 2010). This is supported by behavioural evidence that reward insensitivity is positively correlated with apathy levels in stroke patients (Rochat et al., 2013). Moreover, dopamine plays an important role in the reward network, and methylphenidate, a stimulant whose action results in elevated levels of dopamine in the synaptic cleft, increases activation in the reward network in healthy individuals (Völlm et al., 2004). Methylphenidate has also been shown to reduce apathy after stroke (Martin et al., 1995, Spiegel et al., 2009).

The concept of a reward network has been extensively studied in animals, healthy individuals and individuals with depression, addiction and schizophrenia (Bracht et al., 2015, Cooper, 2002, Haber, 2008, Haber and Knutson, 2010, Lebreton et al., 2009, Mobbs et al., 2003, Nestler and Carlezon, 2006, Rolls, 2000, Sesack and Grace, 2010, Suk Lee et al., 2015). The consensus is that nucleus accumbens and its connections to ventral tegmental area (VTA), amygdala complex, including associated temporal pole, and the prefrontal cortex (particularly medial and orbitofrontal cortex) are the core of the network associated with reward processing (Bracht et al., 2015, Cooper, 2002, Haber, 2008, Haber and Knutson, 2010, Lebreton et al., 2009, Mobbs et al., 2003, Nestler and Carlezon, 2006, Rolls, 2000, Sesack and Grace, 2010, Suk Lee et al., 2015). It is hypothesized that the network is supported by specific fibre bundles, including the cingulum, uncinated fasciculus and medial forebrain bundle and these fibres connecting the nucleus accumbens to the other core brain regions (Von Der Heide et al., 2013, Wu et al., 2016). The nucleus accumbens calculates the probability and magnitude of an expected reward outcome and decides whether to initialize action towards it (Cooper, 2002, Nestler and Carlezon, 2006, Sesack and Grace, 2010). The amygdala complex is associated with positive affect and reinforcement (Haber, 2008, Murray, 2007, Nestler and Carlezon, 2006) whereas the temporal pole relays information between the orbitofrontal cortex and amygdala while being involved in the processing of social rewards (Lebreton et al., 2009, Mobbs et al., 2003). The orbitofrontal cortex is involved in neural representation of primary and complex reinforcers and in controlling and correcting reward behaviour (Haber, 2008, Haber and Knutson, 2010, Lebreton et al., 2009, Nestler and Carlezon, 2006, Rolls, 2000). It is supported in its role by anterior cingulate cortex and medial frontal cortex which are associated with subjective rating of pleasure (Haber, 2008, Haber and Knutson, 2010, Nestler and Carlezon, 2006, Suk Lee et al., 2015).

In this study we use this overall framework to test whether network efficiency, derived from structural MR network analysis is negatively associated with apathy in SVD and whether the reward network plays a key role in mediating this association. To address these questions we apply DTI network analysis in patients with symptomatic SVD and use graph theory to provide a quantitative measure of network efficiency. This technique quantifies how efficient and well-connected the information exchange pathways are within a network. Thus we determine which parts of the network are associated with apathy in SVD, and whether there is selective involvement of the reward network in the association with apathy, as compared with two other “experimental control” networks.

## 2 Materials and methods

### 2.1 Participants

One hundred and twenty one participants with symptomatic SVD were recruited between 2007 and 2010. They were from stroke services at three hospitals covering a geographically contiguous region of South London and recruited as part of the St George's Cognition and Neuroimaging in Stroke (SCANS) study (Lawrence et al., 2013). SVD was defined as a clinical lacunar stroke syndrome (Bamford et al., 1991) with radiological evidence of a lacunar infarct in a brain region consistent anatomically with the clinical syndrome localized in white matter or deep grey matter nuclei and of the size smaller than 1.5 cm, in addition to confluent WMH of Fazekas grade 2 (early confluent) or higher (Fazekas et al., 1987). The inclusion criteria for SCANS was a Fazekas score of 2 or more as determined by the local clinician. All MRIs were then centrally reviewed blinded to clinical, MRI and cognitive details. The Fazekas scores used in the analysis were based on these blinded assessments. Both periventricular and deep WMH were separately rated and an overall score also generated which took into account both periventricular and deep WMH. Exclusion criteria were: any stroke mechanism other than SVD including intra/extra-cranial large artery stenosis > 50%, cardio-embolic source, subcortical infarcts > 1.5 cm in diameter as these are often embolic, or any cortical infarcts; history of major neurological or psychiatric condition excepting depression; non-fluent in English; not suitable for MRI; and unable to give informed consent.

SCANS is a longitudinal prospective study of MRI and cognition in SVD. For this analysis only the baseline measures were used. Out of all the 121 participants included in SCANS study at baseline, seven participants were excluded due to inadequate MRI data (acquisition difficulties or analysis pipeline failure) leaving 114 participants in the final analysis. The study was approved by a local research ethics committee of London–Wandsworth (07/Q0803/82) and all participants were able and provided written informed consent. The capacity of the participants to give informed consent was assessed via the review of their clinical status and by a researcher trained in taking informed consent. The study was registered: www.ukctg.nihr.ac.uk, study ID: 4577 and followed institutional guidelines. The study was conducted in accordance with the Helsinki Declaration as revised in 1989.

### 2.2 Apathy measure

The Geriatric Depression Scale (GDS) (Yesavage et al., 1983) was administered in full and apathy was measured using a subset of six items from this scale. These items were: ‘prefer to stay at home’, ‘avoid social gatherings’, ‘dropped activities and interests’, ‘find life very exciting’, ‘hard to start new projects’ and ‘full of energy’. Responses to these items are ‘yes’ or ‘no’, converted numerically and aggregated to provide an apathy score. The subset of items used have previously been shown to identify apathy, and allow distinction from depressive symptoms, in both healthy older adults (Adams et al., 2004) and SVD (Hollocks et al., 2015), with a meta-analysis concluding that apathy/withdrawal consistently appears as a single distinguishable factor in factorial analyses of GDS in various populations (Kim et al., 2013).

### 2.3 MRI acquisition

The MR acquisition has been described previously (Lawrence et al., 2013). Briefly, all the images were obtained in a 1.5 T General Electric Signa HDxt MRI scanner (General Electric, Milwaukee, WI, USA) with maximum gradient 33mTm^−1^ and a propriety head-coil. All sequences covered the entire brain with a total acquisition time of 45 min. Sequences relevant to the work described here were a T1-weighted sequence using a coronal spoiled gradient recalled echo (SPGR) with 176 1.1 mm thick slices (field of view = 240 × 240 mm^2^, matrix = 256 × 192), TR = 11.5 ms, TE = 5 ms and flip angle of 18°. DTI images were also acquired with an axial single shot spin echo planar sequence comprising 55 slices without any gap (field of view = 240 × 240 mm^2^, matrix = 96 × 96), TR = 15600 ms and TE = 93.4 ms resulting in 2.5 mm^2^ isotropic voxels. First, four images without diffusion weighting (b = 0smm^−2^) were followed by 25 diffusion-weighted (b = 1000smm^−2^) images with gradient applied in non-collinear directions. Subsequently, another four b = 0smm^−2^ images were obtained followed by diffusion weighted images with the negative of the 25 previously acquired directions.

### 2.4 Image pre-processing and network measures

The image analysis methods have been described in detail previously (Lawrence et al., 2014). Briefly, DTI images were eddy current corrected, and a diffusion tensor was fitted to the DTI signal using FMRIB Software Library (FSL) v4.1 (Jenkinson et al., 2012). Whole-brain deterministic diffusion tensor tractography was performed with streamlines terminating at angles of > = 40° between principal eigenvectors in adjacent voxels or at FA< 0.2, which was the threshold relating to the ability of deterministic tensor tractography to give a reliable directional information. Network nodes were defined on the basis of the Automated Anatomical Labelling (AAL) atlas (Tzourio-Mazoyer et al., 2002). For each subject their T1-weighted image was co-registered to their averaged B0 DTI map using FSL FLIRT (Jenkinson et al., 2002, Jenkinson and Smith, 2001). A transformation between the T1-image and MNI space was calculated using Advanced Normalization Tools. These transformations were subsequently applied to the AAL image to transform it into each subject's specific DTI space.

Graph theory describes the brain as nodes or vertices which represent grey matter regions and edges or lines connecting them which represent white matter connections. Such a method of representation helps to quantify mathematical properties of organization in complex neural networks (Bullmore and Sporns, 2009) by allowing to represent potential pathways of information-flow within the network. Thus 90 grey matter nodes were obtained for each subject. Network edges were defined as the presence of streamlines derived from tractography, directly connecting any pair of nodes. Both length (*l*) and number of streamlines (*N*) were taken into account when calculating the edge weighting (*w*_*ij*_) so that the weight of each edge was proportional to the number of its streamlines and inversely proportional to the length of the streamlines. The equation was modified from Hagmann et al. (Hagmann et al., 2007) and was:

$$w_{\mathrm{ij}}=\frac{1}{2}\sum_{m=1}^{N}\frac{1}{l_{m}}$$

Thus undirected weights of edges were obtained for the matrix of 90 × 90 nodes.

### 2.5 Statistical calculations

Network Based Statistics, which is a validated method to perform statistics on large networks, was used to perform the correlation analysis between apathy and connectivity weights of clusters of nodes (Zalesky et al., 2010). Only edges present in at least 30 subjects were taken into account. Mass univariate testing was performed using network based statistics and correlating weights of each edge with apathy levels; edges whose weights correlated with apathy scores at a level of t = 2.5 or higher (corresponding approximately to p < = 0.01 in a two-tailed test) were selected as supra-threshold connections for further analysis. The network based statistics algorithm was then used to extract topological clusters of edges and nodes deemed significantly correlated with apathy in SVD (p < 0.05). Thus, nodes and edges forming a network in which connectivity strength was correlated with apathy levels were identified.

We then investigated whether differences in reward network efficiency were related to variations in apathy. Efficiency of the network is defined as the average value of the shortest inverse path length between each pair of nodes participating in the network. The higher the efficiency of the network, the better-connected the information exchange pathways in the network are. If the efficiency of the network is high the information needs to travel via fewer stages when flowing from node to node. To test the specificity of any association we compared the associations to the degree of variance in apathy explained by two ‘experimental control’ networks, the motor and the visual networks. The three pre-defined sub-networks were delineated from the 90 AAL nodes obtained for each participant. Regions involved in the three networks were based on meta-analyses and review of papers of functional MRI and tractography studies in healthy individuals and individuals with depression (Bracht et al., 2015, Von Der Heide et al., 2013, Wu et al., 2016).

The networks were delineated as follows:

a) Reward: left and right anterior cingulate cortex (frontal part of cingulum bundle), left and right putamen (nucleus accumbens incorporated), left and right inferior orbitofrontal cortex, left and right medial orbitofrontal cortex, left and right superior medial cortex, right and left amygdala, right and left superior temporal pole, right and left middle temporal pole (uncinate fasciculus)
b) Motor: left and right precentral cortex, left and right supplementary motor area, and left and right putamen
c) Visual: left and right superior occipital cortex, left and right middle occipital cortex, left and right inferior occipital cortex, left and right fusiform gyrus, and left and right parietal superior cortex

The efficiency of connectivity within each of these three networks was calculated using the weights of all possible edges between the nodes within the network, with the Brain Connectivity Toolbox (Rubinov and Sporns, 2010). The efficiency of each network was correlated with the apathy scores (p < 0.05 considered as significant). To test whether efficiency of any of the networks explained additional variance in apathy levels relative to the two other networks, we performed partial correlation between apathy and efficiency of each of the networks while controlling for efficiency in each of the other networks. The partial correlation was regarded as significant if the p-value was < 0.05. Since the network efficiency is affected by possible disconnections, we repeated the analysis with the use of sum of weights instead of efficiency of the a-priori sub-networks. The same significance levels were applied in this analysis.

### 2.6 Controlling for age, vascular risk factors and SVD burden

Additionally, since age and vascular risk factors, that is hypertension (defined as systolic blood pressure > 140 mmHg or diastolic blood pressure > 90 mmHg or on hypertensive medication), hypercholesterolemia (defined as on statins therapies or a total cholesterol > 5.2 mmol/l), diabetes mellitus (clinical diagnosis) and smoking (defined as current smoking), are important determinants of SVD we tested whether controlling for these risk factors changed the significance of the correlation between apathy and efficiency of the three neural networks. To achieve this goal we used partial correlation with the p-value < 0.05 deemed as significant.

We also tested whether apathy correlated with one of radiological markers of the severity of SVD. To do this we determined WMH volume, number of lacunas, number of cerebral microbleeds and number of perivascular spaces, using methods previously described (Benjamin, 2018, Benjamin et al., 2016, Lawrence et al., 2013). Briefly, WMH volume was calculated based on single-rater delineation of lesions on FLAIR images in the semi-automated DISPUNC program. Number of lacunas was determined on the basis of T1-weighted and FLAIR images, where a lacuna was defined as a corticospinal fluid filled cavity within the white matter or subcortical regions, between 3 and 15 mm in diameter. Number of microbleeds was quantified on the basis of gradient echo images, where a microbleed was a well-defined focal area of low signal < 10 mm in diameter. Number of perivascular spaces was calculated on the basis of T2 images where a perivascular space was defined as a smaller than 3 mm punctuate or linear hyperintensity in basal ganglia or centrum semiovale. To do all this we performed correlations which were regarded significant if the p-value was < 0.05.

## 3 Results

Demographic and clinical variables of the study population are shown in Table 1. The mean age was 70 years (SD = 9.6 years), and 75/114 (65.8%) were male.

**Table 1:** Demographic and clinical variables of participants with SVD in the final analysis.

Number of participants	114
Mean age in years (SD)	70.0 (9.6)
Gender male (%)	75 (65.8%)
Hypertension (%)	101 (88.6%)
Systolic BP (SD)	147.16 (21.5)
Diastolic BP (SD)	81.06 (10.7)
Hypercholesterolemia (%)	104 (91.2%)
Diabetes mellitus (%)	22 (19.3%)
Current or ex-smoker (%)	63 (52.6%)
BMI kg/m^2^ (SD)	26.9 (4.8)
Fazekas Scale (%)	Fazekas 3 – 31 (27.2%)
	Fazekas 2 – 66 (57.9%)
	Fazekas 1 – 17 (14.9%)
Apathy (SD)	2.9 (1.7)

**Fig. 1:**
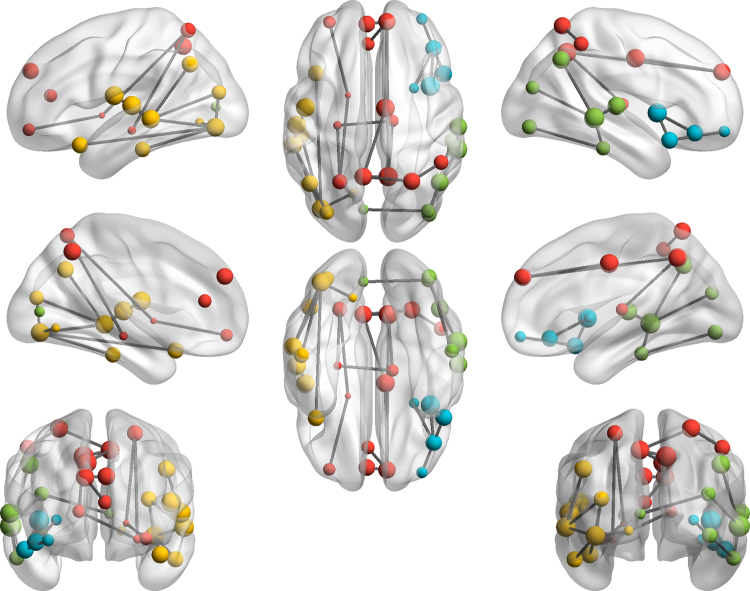
Topological clusters showing significant negative correlation between apathy and connectivity strength on the level p < 0.05 (corrected for multiple comparisons) in the whole brain analysis. Different colours represent the four clusters whose neural connectivity correlates negatively with apathy. The nodes forming the clusters are described in Table 2 (cluster 1 = red; cluster 2 = yellow; cluster 3 = green; cluster 4 = blue). (For interpretation of the references to color in this figure legend, the reader is referred to the web version of this article.)

Fig. 1 illustrates topological clusters in which connectivity strength correlated negatively with apathy. The clusters encompassed medial frontal lobes, basal ganglia, parietal lobes and temporal nodes. Table 2 presents the p-values of the correlation in each of the four observed clusters, the number of edges and nodes involved and the location of the nodes that participate in it. Some of the nodes observed in these clusters, such as the putamen, anterior cingulate cortex or inferior temporal pole, are considered to be part of the reward network, although the clusters also encompass other nodes which would not be classified as such, e.g. the precuneus or angular gyrus. In contrast to these negative correlations, there were no positive correlations with apathy, suggesting that loss of connectivity is always associated with higher apathy.

**Table 2:** Nodes participating in the topological clusters showing a significant negative correlation between the connectivity strength and apathy levels in the whole brain analysis (p < 0.05).

Topological cluster	Edges count	Nodes count	Nodes participating	P-value
Cluster 1	13	13	L middle orbitofrontal cortex	P < .001
			L&R medial superior frontal	
			L anterior cingulate cortex	
			L pallidum	
			R middle cingulate cortex	
			L hippocampus	
			R thalamus	
			L&R superior parietal cortex	
			L&R precuneus	
			R inferior parietal cortex	
Cluster 2	9	9	L superior temporal pole	P = .002
			L rolandic Operculum	
			L superior temporal cortex	
			L inferior temporal cortex	
			L middle temporal cortex	
			L angular gyrus	
			L lingual gyrus	
			L middle occipital cortex	
			L inferior occipital cortex	
Cluster 3	6	7	R superior temporal cortex	P = .009
			R inferior temporal cortex	
			R middle temporal cortex	
			R angular cortex	
			L calcarine fissure	
			R middle occipital cortex	
			R inferior occipital cortex	
Cluster 4	4	5	R middle orbitofrontal cortex	P = .039
			R inferior orbitofrontal cortex	
			R superior temporal pole	
			R insula	
			R putamen	

The efficiencies of the three chosen sub-networks all correlated negatively with apathy scores; r = −0.344 for the reward network (p < 0.001), r = −0.233 for the motor network (p = 0.013) and r = −0.210 for the visual network (p = 0.025). The sums of weights of the three sub-networks were also all negatively correlated with the apathy scores s: r = −0.372 for the reward network (p < 0.001), r = −0.250 for the motor network (p = 0.007) and r = −0.210 for the visual network (p = 0.025).

The partial correlation calculation showed that variability in the reward network efficiency explained additional variance in apathy when the correlation was controlled for efficiency in the other two networks. The reverse was not observed: that is, variability in efficiencies of the visual and motor network did not explain significant additional variance in apathy when the correlations were controlled for the effect of reward network. The partial correlation values and the corresponding p-values are presented in Table 3. The sums of weights within the sub-networks followed the same pattern, and their partial correlation values and corresponding p-values are also presented in Table 3.

**Table 3:** Partial correlation between efficiency of a-priori defined sub-networks and apathy levels with the contribution of the remaining networks controlled.

Network correlated	Control network	Apathy correlation value when controlled for other networks		p-value (two-tailed) when controlled for other networks	
		Efficiency	Sum of weights	Efficiency	Sum of weights
Reward network	Visual network	−0.289*	−0.323*	P = .002	P<.001
	Motor network	−0.27*	−0.293*	P = .004	P = .002
Motor network	Reward network	−0.074	−0.075	P = .439	P = .432
	Visual network	−0.177	−0.203*	P = .06	P = .031
Visual network	Reward network	−0.08	−0.051	P = .401	P = .588
	Motor network	−0.145	−0.136	P = .125	P = .151

* Significant partial correlations (p < 0.05).

The significance of the correlation between apathy and efficiency of the three neural networks did not change when age and vascular risk factors were controlled for. When controlled for these variables simultaneously apathy levels significantly correlated with efficiency of the reward network (r = −0.355, p-value < 0.001), motor network (r = −0.234, p-value = 0.014) and visual network (r = −0.206, p-value = 0.031).

Apathy did not correlate significantly with WMH volume (r = 0.122, p-value = 0.198), number of lacunas (r = 0.169, p-value = 0.072), number of microbleeds (r = 0.139, p-value = 0.141) or number of perivascular spaces (r = 0.072, p-value = 0.445).

In summary, this analysis shows that although the efficiencies of all three networks are mutually correlated, the efficiency of the reward network explains variability in apathy levels that is not explained by the other networks, risk factors or radiological markers of SVD severity.

## 4 Discussion

In our study we identified clusters of nodes whose connectivity is correlated with apathy in SVD. Among these nodes there were also some associated with reward processing. Subsequently, in our study we found that the efficiency of the reward network was negatively correlated with levels of apathy in individuals with SVD. Variability in reward network efficiency explained additional variance in apathy levels that was not explained by the efficiencies of two control networks, the motor and visual networks. This variability was also not explained by differences in age, vascular risk factors of severity of radiological markers of SVD. These results suggest white matter pathways underlying the reward networks are associated with increased levels of apathy in SVD.

The reward network has been previously associated with motivation and its disorders in healthy individuals and in patients (Barch et al., 2015, Bressan and Crippa, 2005, Suk Lee et al., 2015). Lesion studies in stroke suggest that damage to the structures pivotal for motivation and reward processing in humans, particularly the frontal lobes and basal ganglia, result in apathy (Grool et al., 2012, Grool et al., 2013, Gupta et al., 2014, Hama et al., 2011, Yang et al., 2013). Changes in white matter integrity have been previously associated with apathy in SVD (Hollocks et al., 2015). Our results provide further insight as to why apathy occurs to variable degrees in SVD. They not only emphasise the importance of white matter integrity, but also provide novel information on the specific neural localization and function of motivation and reward. Thus the results of this analysis suggest a united psychological and neurobiological explanation of the phenomenon. They also emphasise a key role of the reward network in development or maintenance of apathy in SVD.

The findings may have implications for the treatment of apathy in SVD. Dopamine is an important neurotransmitter responsible for pharmacological connectivity within the human reward network. Increasing levels of dopamine could be a potential target for treatment of apathy in SVD. Initial pilot data towards such a treatment in patients suffering from stroke are promising, including medication such as methylphenidate or Ropinirole (Kohno et al., 2010, Martin et al., 1995, Spiegel et al., 2009).

## 5 Limitations

A potential limitation of the analysis is that all of the described network measures refer exclusively to the cerebrum, including the associated cerebral cortical and subcortical structures. The cerebellum was not included in the tractography analysis as there was a significant minority of participants whose field of view did not cover this region. Regions of the brain stem were also excluded because of the small size and a lack of T1 weighted images contrast relative to surrounding structures. Thus the nodes described here did not contain VTA which plays an important role in reward processing, nor parts of the cerebellum participating in motor control. In this paper we only test for the linear correlation between apathy and efficiency of the delineated networks. However, further exploration may be beneficial as to the linearity of the correlation.

## 6 Conclusion

In summary, this work implicates the importance of disruption of the reward network in determining whether patients with SVD suffer apathy. Treatments which improve connectivity in this network may improve apathy in SVD.
